# Genome analysis of haloalkaline isolates from the soda saline crater lake of Isabel Island; comparative genomics and potential metabolic analysis within the genus *Halomonas*

**DOI:** 10.1186/s12864-023-09800-9

**Published:** 2023-11-20

**Authors:** Luis Mario Hernández-Soto, Francisco Martínez-Abarca, Hugo Ramírez-Saad, Marcos López-Pérez, José Félix Aguirre-Garrido

**Affiliations:** 1 Doctorado en Ciencias Biológicas y de La Salud, Universidad Autónoma Metropolitana, Mexico City, Mexico; 2 Estructura, Dinámica y Función de Genomas de Rizobacterias, Departamento de Microbiología del Suelo y Sistemas Simbióticos, Estación Experimental del Zaidín-CSIC, Granada, Spain; 3 Departamento Sistemas Biológicos, Universidad Autónoma Metropolitana-Xochimilco, Ciudad de Mexico, México; 4 Departamento de Ciencias Ambientales, Universidad Autónoma Metropolitana-Lerma, Estado de México, Lerma, México

**Keywords:** Genome mining, *Halomonas*, Halophilic lake, Metabolic capacities, Mexican lake

## Abstract

**Background:**

Isabel Island is a Mexican volcanic island primarily composed of basaltic stones. It features a maar known as Laguna Fragatas, which is classified as a meromictic thalassohaline lake. The constant deposition of guano in this maar results in increased levels of phosphorus, nitrogen, and carbon. The aim of this study was to utilize high-quality genomes from the genus Halomonas found in specialized databases as a reference for genome mining of moderately halophilic bacteria isolated from Laguna Fragatas. This research involved genomic comparisons employing phylogenetic, pangenomic, and metabolic-inference approaches.

**Results:**

The Halomonas genus exhibited a large open pangenome, but several genes associated with salt metabolism and homeostatic regulation (*ect*ABC and *bet*ABC), nitrogen intake through nitrate and nitrite transporters (*nas*A, and *nar*GI), and phosphorus uptake (*pst*ABCS) were shared among the *Halomonas* isolates.

**Conclusions:**

The isolated bacteria demonstrate consistent adaptation to high salt concentrations, and their nitrogen and phosphorus uptake mechanisms are highly optimized. This optimization is expected in an extremophile environment characterized by minimal disturbances or abrupt seasonal variations. The primary significance of this study lies in the dearth of genomic information available for this saline and low-disturbance environment. This makes it important for ecosystem conservation and enabling an exploration of its biotechnological potential. Additionally, the study presents the first two draft genomes of *H. janggokensis*.

**Supplementary Information:**

The online version contains supplementary material available at 10.1186/s12864-023-09800-9.

## Introduction

Isabel Island (or Isabela Island) has a volcanic origin and is mainly composed of basaltic stone [1]. Its geographic location is 21.846621 N, 105.883377 W, and it is considered a continental island, although it is located ~30 km from San Blas port in Nayarit, Mexico [2]. Within the exposed surface, there are numerous scattered craters that originated from ancient explosions [3]. The most remarkable and well-preserved one hosts a small circular (maar) lake called 'Lago Crater' or 'Laguna Fragatas' [4]. This lake has a diameter of 160 m. It’s classified as a meromictic thalassohaline lake [5], with some fluctuations in its water levels depending on the rains and less probably related to the communication through porous rocks with seawater [5]. The lake does not appear to have a direct connection to the ocean, as it does not display fluctuations corresponding to marine tides [6]. As with most thalassohaline lakes, the dominant ions found in Laguna Fragatas are sodium and chloride, similar to those found in the ocean [7]. The exposed rocks lining the walls of the lake have likely undergone alterations as a result of the annual fluctuations in water levels or due to the chemical effects of guano. Isabel Island is home to numerous marine birds, and their excrement, plays a significant role in the lake's ecosystem. The guano serves as the primary source of carbon, nitrogen, and phosphorus for the lake community [8, 9]. Due to the significant presence of seabirds and the ecological importance of the island, the Mexican government declared Isabel Island a National Park (Ecological Reserve) in 1980 [10]. As part of an environmental management plan implemented in 2006, human activities on the island were restricted to ecotourism and responsible fishing [11] . These measures further diminished the already minimal human impact on the lacustrine ecosystem of Laguna Fragatas, ensuring its preservation and protection.

Hypersaline environments have salt concentrations higher than regular seawater (greater than 0.6 M). Halophiles organisms are microorganisms capable of live in hypersaline environments and often require a high salt concentration for their growth [12]. In 1978, Kushner [13] proposed an internal division based on the amount of salt they required for proper development, categorizing them as slight, moderate, and extreme halophiles. These microorganisms can be found in all three domains of life and are distinguished by their requirement of high salinity conditions for growth [14, 15]. Halophiles, halotolerant, and non-halophilic organisms can be closely related in phylogenetic trees. Despite this heterogeneity, some phylogenetically coherent groups include only halophile organisms. Archaea belonging to the class Halobacteria and Bacteria in the order Halanaerobiales, or family Halomonadaceae are examples of taxonomic groups comprising only these organisms. *Halomonas* is the type genus of the family Halomonadaceae, and *H. elongata* is the type species of the genus [16]. It is not monophyletic and comprises two separated phylogenetic groups containing many species [17]. It was proposed as a genus in 1980 [18]. The members of this genus have been used as models for studies of halophily. They grow better aerobically, but some species can grow using nitrate, nitrite, or fumarate as an electron acceptor in the presence of glucose [19]. Some of their representatives are highly halophilic bacteria [20], adapted to a wide range of saline concentrations [21]. Despite these common characteristics, the genus has other heterogeneous features shared among its members, and some of its species have promising industrial uses, as the production of betaine, ectoine, polyhydroxyalkanoates, biosurfactants, among others and the bioremediation of industrial wastes [22]. The mining of complete genomes from isolated halophile organisms allows for the identification of previously uncharacterized biosynthetic gene clusters within the genomes of sequenced organisms [23] and facilitates the understanding of the environmental dynamics within those halophilic sites. This process involves not only the computational prediction of biosynthesis-related genes but also functional interrogation, ideally leading to a comprehensive understanding of the related chemistry [24].

The main relevance of this study lies in the lack of genomic information from organisms isolated from this low disturbed hypersaline environment. The analysis utilizes high-quality genomes from *Halomonas* strains deposited in the NCBI database, with KEGG database serving as a guide (due to its thorough curation, substantial number of entries, and citations) for genome mining of the moderate halophilic strains genomes (Hven4, Hven7, Hven9, Hven10, Hjan13, and Hjan14) isolated from Laguna Fragatas by Aguirre [25] between 2016 and 2020 for this. This work also reports the first two draft genomes of strains from *H. janggokensis*.

## Materials and methods

The genomic material used in this study came from bacteria isolated by Aguirre-Garrido et al. [26] (*H. venusta* strains Hven4, Hven7, Hven9, *H. janggokensis* Hjan13, and Hjan14), *H. venusta* Hven10 was isolated specifically for this study. The genome from *H. venusta* DSM 4347^ T^ was originally isolated from marine water in Hawaii, and its genome was assembled and annotated by Martinez-Abarca, et al. [27]. Briefly, the strains were cultivated in LBS10 medium and were identified by 16S rRNA gene Sanger sequencing to [26].

### Genome sequencing

Whole-genome shotgun sequencing of genomic DNA was done at the Integrated Microbiome Resource (IMR) from Dalhousie University, Canada. The libraries were prepared using the Illumina Nextera Flex Kit for the MiSeq platform (150 + 150 bp PE) [28]. The quality of the obtained sequences was checked using Fastqc 0.11.9 [29]. Reads with qualities lower than Phred 20 and lengths smaller than 280 bp were removed using Trimmomatic 0.38 [30]. Genome assemblies were done with Unicycler [31] enhancing SPAdes [32]. The quality of the assembled genomes was calculated using QUAST [33]. Contigs smaller than 1000 bp were discarded as they could lead to mismatched assemblies, and bigger ones were mapped to the same species reference genome against *H. venusta* DSM 4743^ T^, Hjan strains have no reference genome available using Geneious mapper [34]. Contigs were visualized with Mauve to check the order of the genomes due to its capability to leverage synteny to facilitate the analysis of genome alignments [35]. The genomes assembled and the database obtained were graphically represented utilizing BRIG [36].

### Functional annotation and pangenome analysis

The assembled genomes from our isolates were compared against a set of complete genomes of *Halomonas* spp. downloaded from the NCBI database [37] using KEGG (Kyoto Encyclopedia of Genes and Genomes) database as a guide for selecting genomes with known metabolic pathways (Table 1). The annotation of our assembled genomes was carried out using the NCBI’s Prokaryotic Genome Annotation Pipeline (PGAP) [38]. Annotated genomes were used as input to obtain the pangenome with Roary 3.13.0 [39, 40] and Anvio 7.1 [41] by a Diamond alignment [42]. Anvi’o was also used for inferring the metabolic pathways modules compared against KEGG [43–45]. Data was organized, visualized, and plotted using R [46], Tidyverse [47], and ggplot2 [48]. TRIBE-MCL algorithm, based on Markov clustering [49] for the assignment of proteins into families based on pre-computed sequence similarity information is the approach used by Roary and Orthofinder-tools package [39, 50], for calculating the relationships between true orthologs genes (OG). Roary makes a classification into core and accessory genomes. The core genes present in the genomes are further divided into hard-core (present in > 99% of genomes) and soft-core (present in 95–99% of genomes) genes. Additionally, there are shell genes (found in 15–95% of genomes) and cloud genes (present in less than 15% of genomes) that comprise the accessory genome [51]. For the phylogenetic analyses, ANI values were calculated using Anvi’o 7.1 [52] via pyANI [53, 54]. OrthoANI was used for comparing the genomic similarity between the coding regions of the genomes [55].

**Table 1:** *Halomonas* genomes available in the GenBank database used in this study, including their basic statistical information. The list is ordered by G + C content (mol%). Strains sequenced in this study, represented in bold, correspond to isolates from Laguna Fragatas. Hven = *H. venusta,* Hjan = *H. janggokensis*

Strain designation	Source	Accession number	Size (Mb)	GC (mol%)	CDS	tRNAs	Contigs	N50 Length (Mb)	Drafts ≥ N50	Coverage (x)	Sequencing technology	Genome status
*H. beimenensis* NTU-111	Solar saltern (Taiwan)	CP021435.1	4.05	68.4	3,686	76	1	4.05	1	160	PacBio	Complete
*H. aestuarii* Hb3^T^	Solar saltern (Korea)	CP018139.1	3.54	67.9	3,236	70	1	3.54	1	100	PacBio	Complete
*H. campisalis* SS10-MC5^T^	Alkaline lake (USA)	CP065435.1	4.44	66.1	4,056	69	47	252.6	1	275	Illumina	Draft
*H. sulfidoxydans* MCCC 1A11059^T^	Surface sediment (China)	CP053381.1	4.49	66.0	4,071	69	1	4.49	1	382	PacBio	Complete
*H. sulfidivorans* MCCC 1A13718^T^	Deep sea sediment (Pacific Ocean)	CP053383.1	4.57	64.2	4,198	69	1	4.57	1	576	PacBio	Complete
*H. elongata* DSM 2581^ T^	Solar salt (Netherlands Antilles)	FN869568.2	4.06	63.6	3,739	70	1	4.06	1	17.4	Roche 454	Complete
*H. chromatireducens* AGD 8–3	Soda soil (Russia)	CP014226.1	3.97	62.8	3,679	61	1	3.97	1	34	Illumina	Complete
*H. hydrothermalis* Y2	Pulp mill wastewater (China)	CP023656.1	3.93	60.2	3,573	62	1	3.93	1	34	Illumina	Complete
*H. huangheensis* BJGMM-B45^T^	Soil (China)	CP013106.1	4.76	58.5	4,142	65	1	4.76	1	317	Illumina/ PacBio	Complete
*H. meridiana* SCSIO 43005^ T^	Coral reef (China)	CP024621.1	3.86	58.1	3,628	61	1	3.86	1	100	PacBio	Complete
*H. piezotolerans* NBT06E8^T^	Deep sea (Marshall Islands)	CP048602.1	3.95	57.9	3,595	61	1	3.95	1	212	PacBio	Complete
***H. janggokensis* Hjan13**	Saline lake (Mexico)	JAKNQU000000000	3.99	57.4	3,647	57	19	725,804	3	90	Illumina	Draft
***H. janggokensis* Hjan14**	Saline lake (Mexico)	JAKNQT000000000	3.99	57.4	3,647	57	19	709,144	3	92	Illumina	Draft
*H. axialensis* Althf1^T^	Sulfide rock (North America Pacific Ocean)	AP019517.2	3.62	56.8	5,604	62	1	3.62	1	100	Oxford Nanopore	Complete
*H. olivaria* TYRC17^T^	Salted olive processing effluents (Morocco)	AP019416.1	5.00	55.3	7,586	61	1	5.00	1	100	Oxford Nanopore	Complete
*H. titanicae* SOB56	Sediment (China)	CP059082.1	5.28	54.6	4,839	62	1	5.28	1	100	Illumina	Complete
*H. sulfidaeris* ATCC BAA-803^ T^	Metal sulfide rock (South Pacific Ocean) 1P	AP019514.1	4.21	53.8	7,107	62	2	4.2	2	100	Oxford Nanopore	Complete
***H. venusta* Hven4**	Saline lake (Mexico)	CP090950	4.27	52.6	3,913	61	26	1	2	65	Illumina	Draft
***H. venusta* Hven7**	Saline lake (Mexico)	CP090951	4.27	52.7	4,115	62	27	0.88	3	94	Illumina	Draft
***H. venusta* Hven9**	Saline lake (Mexico)	CP090952	4.27	52.7	3,977	58	26	0.40	4	56	Illumina	Draft
***H. venusta* Hven10**	Saline lake (Mexico)	JAUYZU000000000	4.47	52.8	4,156	30	36	0.25	7	57	Illumina	Draft
*H. venusta* DSM 4743^ T^	Seawater (USA)	CP066539.1	4.28	52.8	3,816	60	3	4.28	1	40	Illumina	Draft
*H. alkaliphila* X3	Marine cage sediment mud (China)	CP024811.1	4.03	52.7	3,636	60	1	4.03	1	100	Complete Genomics	Complete
*H. campaniensis* LS21	Sludge of a saline lake (China)	CP007757.1	4.07	52.6	3,656	60	1	4.07	1	200	Illumina	Complete

## Results and discussion

### Genome sequencing of five *Halomonas* isolates

The six genomes got assembled successfully, getting complete genomes for Hven4, Hven7, Hven10, and Hven9; and draft genomes for Hjan13 and Hjan14. Quality parameters of all the assembled genomes are show in Table 1. N50 values are less than 5 contigs, and the gotten coverages oscillated from 56 × to 94x. The length of the genomes was around 4 Mb (Table 1), coinciding with the length of the *Halomonas* genomes, which fluctuated from 3.5 to 5 Mb. The genomes are graphically represented in Supplementary Fig. 1 indicating the GC distribution and GC skew. The focus was specially directed towards *H. janggokensis* due to the lack of previous records regarding the complete genome sequencing of the type strain M24^T^ [56] This study marks the initial stride in offering genomic information concerning *H. janggokensis*.

### Phylogenetic analysis of *Halomonas* spp. reveals major subdivisions inside the genus

*Halomonas* genomes obtained and assembled were compared against the highest quality genomes available in databases to assure the reliability of the comparations. This was particularly important to improve the genomic mining necessary for further comparisons. Members of the genus *Halomonas* exhibit moderate halophilicity. While certain individuals within this genus may employ metabolisms involving nitrate, nitrite, and other electron acceptors, the majority of their species demonstrate a chemoorganotrophic metabolism [15]. Conversely, in other saline lakes, microbial communities are dominated by other *Arthrospira* [57], *Burkholderia* [58], and *Pseudoalteromonas* [59]. *Halomonas* has also been identified as a significant source of carbohydrate-degrading enzymes from soda lakes in Ethiopia [60]. Sorokin et al. [61] suggest that several members of the genus *Halomonas* are the most metabolically diverse in soda lakes, in addition to having the capability to fully mineralize Glycine Betaine, which is one of the most widely used osmolytes by halophilic bacteria.

**Fig. 1:**
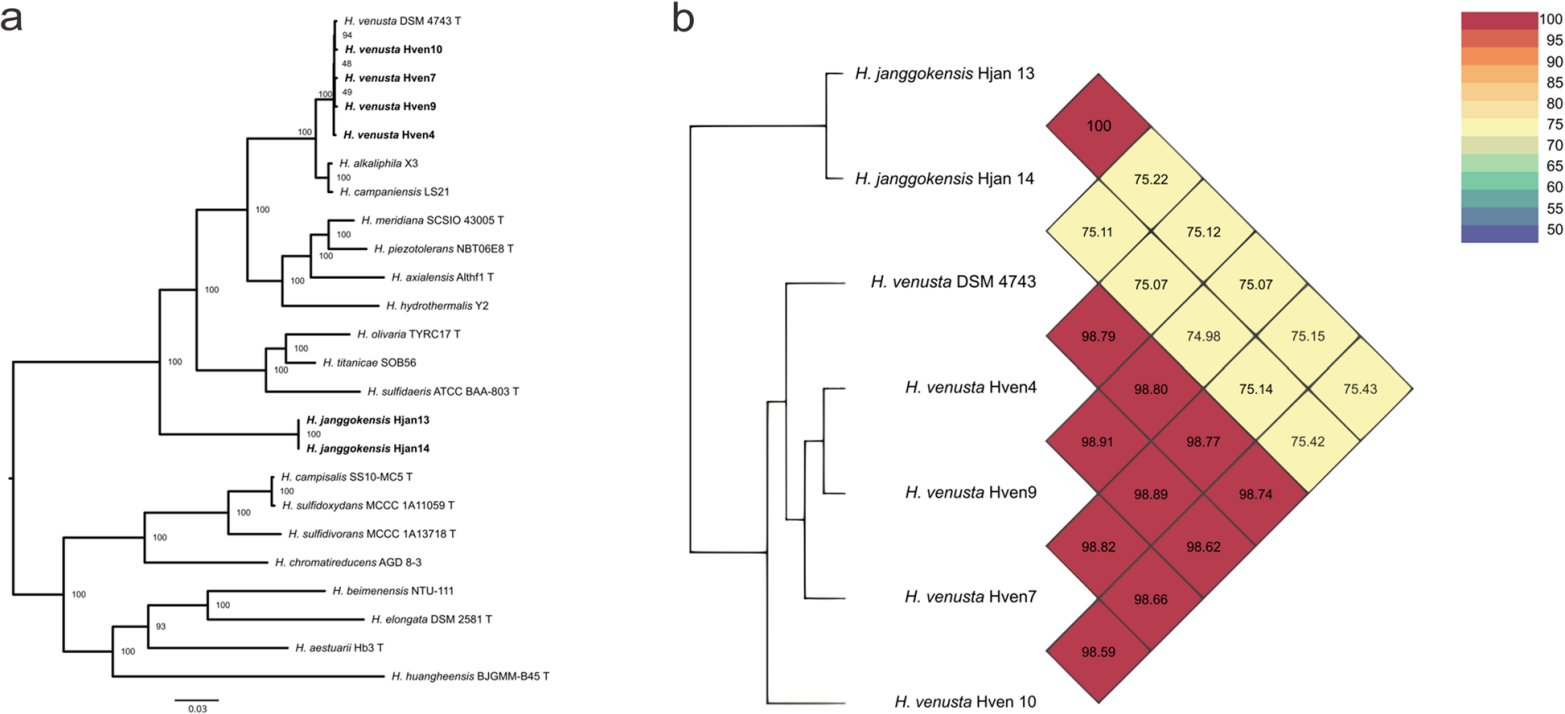
**a** Phylogenetic tree of the 24 *Halomonas* strains studied whose genomes are available in metabolic pathways studies in KEGG. The tree was generated using the average nucleotide identity (ANI) values. It shows the phylogenetic relationships of the isolated strains among the *Halomonas* genus. **b** Average nucleotide sequence identity analysis shows them to belong to the same species. Identity percentages above 70% indicates affiliation to the same genus; and 95 to 100% identity to the same species

An average nucleotide identity (ANI) tree was constructed showing the phylogenetic relationship of Laguna Fragatas among the *Halomonas* selected genomes (Fig. 1a). The tree has two main clades with six different phylogroups. Phylogroup I, include *H. alkaliphila*, *H. campaniensis*, all the group of *H. venusta* isolated in this study, and its type strain DSM 4743^ T^. Phylogroup II includes *H. axialensis*, *H. meridiana*, and *H. piezotolerans*, and *H. hydrothermalis* well known for sharing the capability of living in high deep oceans dealing with high pressures in salty environments [62–64]. Phylogroup III relates *H. olivaria*, *H. titanicae*, *H. sulfidaeris,* and the pair of *H. janggokensis* isolated from Isabel Island Lake; in this comparison level, those isolates seem to be more related to each other than to *H. venusta* from phylogroup II. Phylogroup IV showed a closed relationship between *H. campisalis, H. sulfidivorans*, *H. sulfidivorans*, *H. chromatireducens* and *H. sulfidoxydans* strains reported to have metabolisms specialized in sulfur bioconversion [65]. Finally, *H. huangheensis* and did not fully integrate into its own phylogroup. In addition, the phylogenetic tree showed distant species, including *H. aestuari*, *H*. *beimeninsis*, and *H. elongata*.

Within the phylogroup isolated from the crater, *H. venusta* strains Hven4 and Hven9 showed a closer relationship based on average nucleotide identity, comparing similarity between the coding regions of the genomes. Despite belonging to the same species, strains Hven9 and Hven10 have a slightly divergence. Hjan strains are completely related each other and the discrepancy against Hven strains is notable (Fig. 1b).

### The *Halomonas* pangenome is large and open

**Fig. 2:**
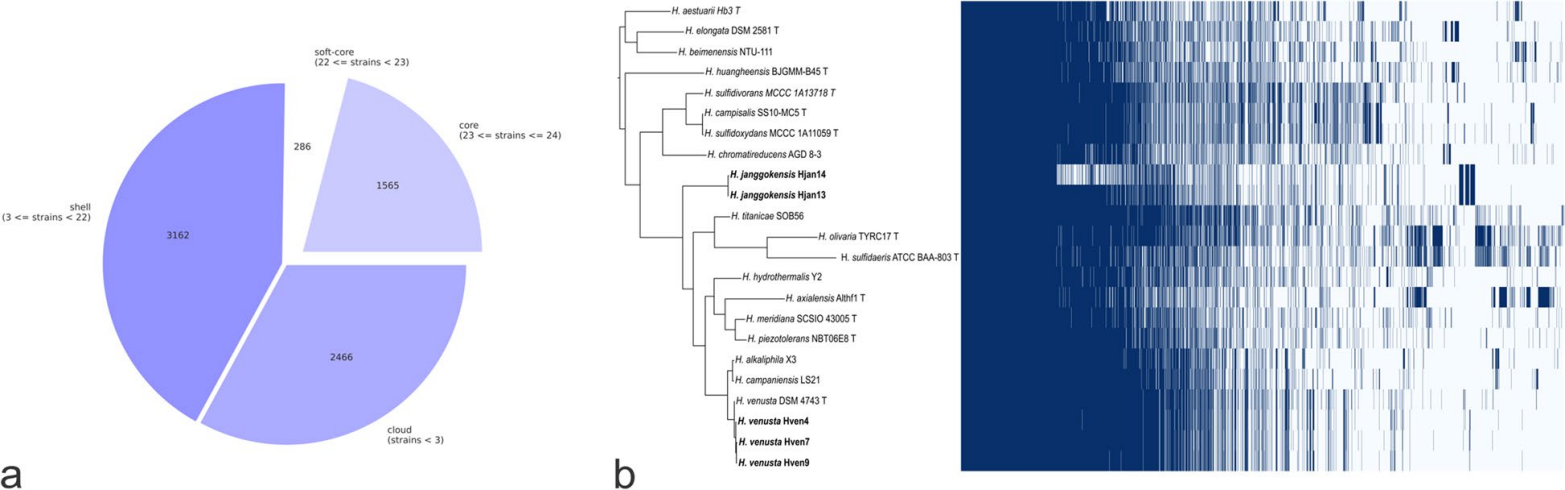
The Pangenome of *Halomonas* genus. **A** A pie chart representation of the pangenome of 24 *Halomonas* strains is cited in Table 1. The chart shows the proportion of genes classified according to their presence within the genus. Core genes are found in > 99% of genomes, soft core genes are found in 95–99% of genomes, shell genome are found in 15–95%, and cloud genome are present in less than 15% of genomes. **B** Gene presence/absence matrix from pangenome analysis of 24 *Halomonas* strains. Each row shows each isolate's gene profile and how conserved the Halomonas core genome is

This analysis defined the size of the pangenome increases steadily with the addition of each other genome suggesting *Halomonas* has a large, open pangenome. This was based on the translated amino acid sequence comparison of the 24 analyzed genomes. The pangenome comprises 100,043 genes gathered in 7479 orthogroups (OGs), representing 95% of the total. From those, 1565 OGs are present in the hard-core and 286 in the soft-core, a conservative portion of the pangenome. Meanwhile, the shell genome has 3162 OGs, and the cloud genome has 2466 (Fig. 2a). Hven isolates and the type strain showed a robust core genome having slight changes located mainly in the cloud genome. Meanwhile, despite the lack of information, Hjan13 and Hjan14 showed specific metabolic signatures in the cloud genome and some concordance between the core genomes (Fig. 2b). The identified signatures are depicted in the metabolic pathways involved in the biosynthesis of terpenes and isoprenoids with chain lengths ranging from 5 to 20 carbons, as illustrated in Fig. 7.

### *Halomonas* genomes have substantial and diverse biosynthetic potential

**Fig. 3:**
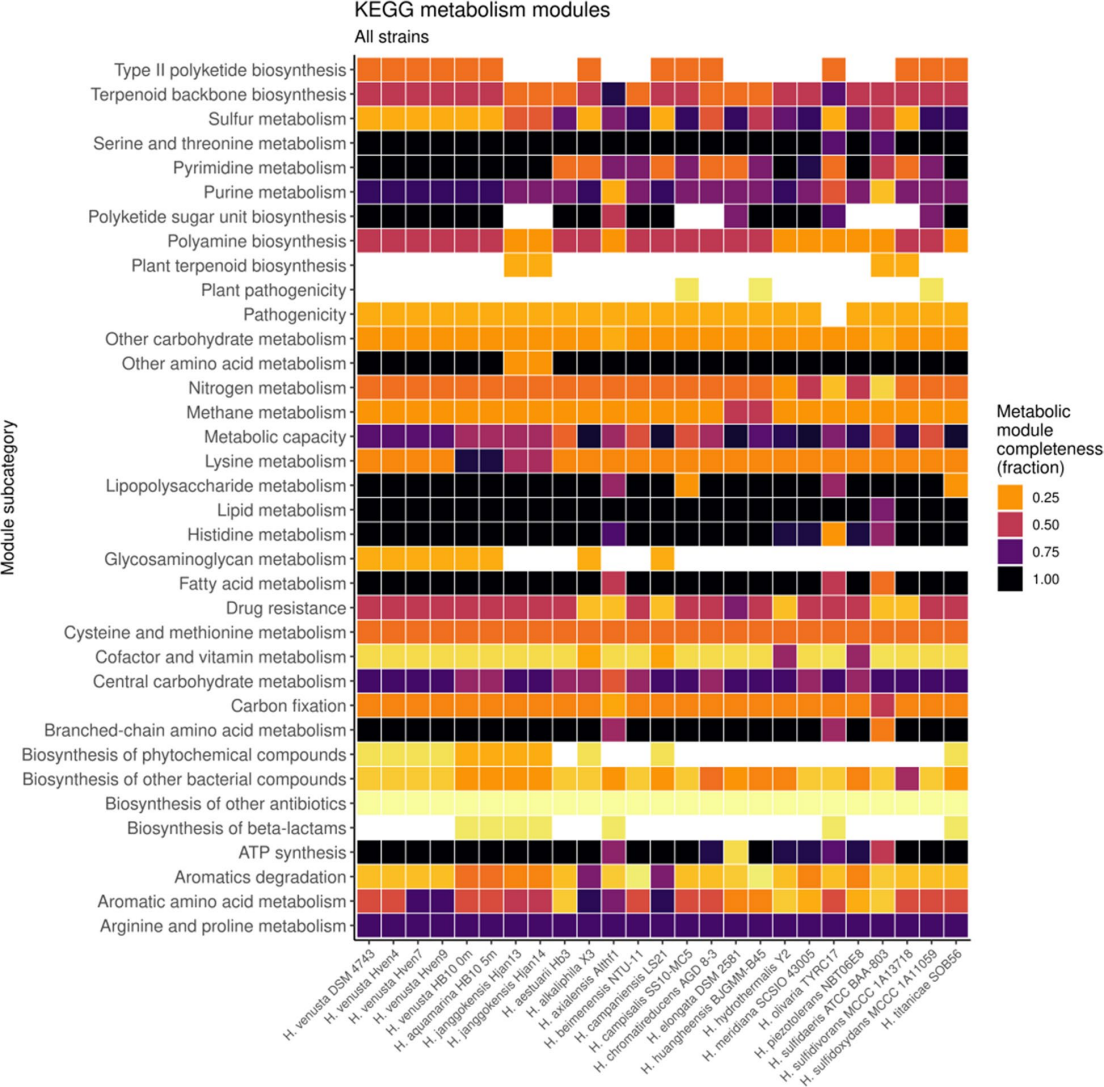
Heatmap showing KEGG modules categories and subcategories found in the complete genomes. Each row represents one KEGG Orthologous module. Completeness higher than 0.70 is necessary to assume that the module has the number of genes to be considered functional. Darker squares show more density of genes

The metabolic functions assigned according to the KEGG modules are shown in Fig. 3. There are differences between species and completeness of the modules showing the calculated metabolic capabilities of each one; those capabilities are closely related to the developed mechanisms for adaptation to the environmental selection pressures present in their natural environments.

### Osmotic regulation

**Fig. 4:**
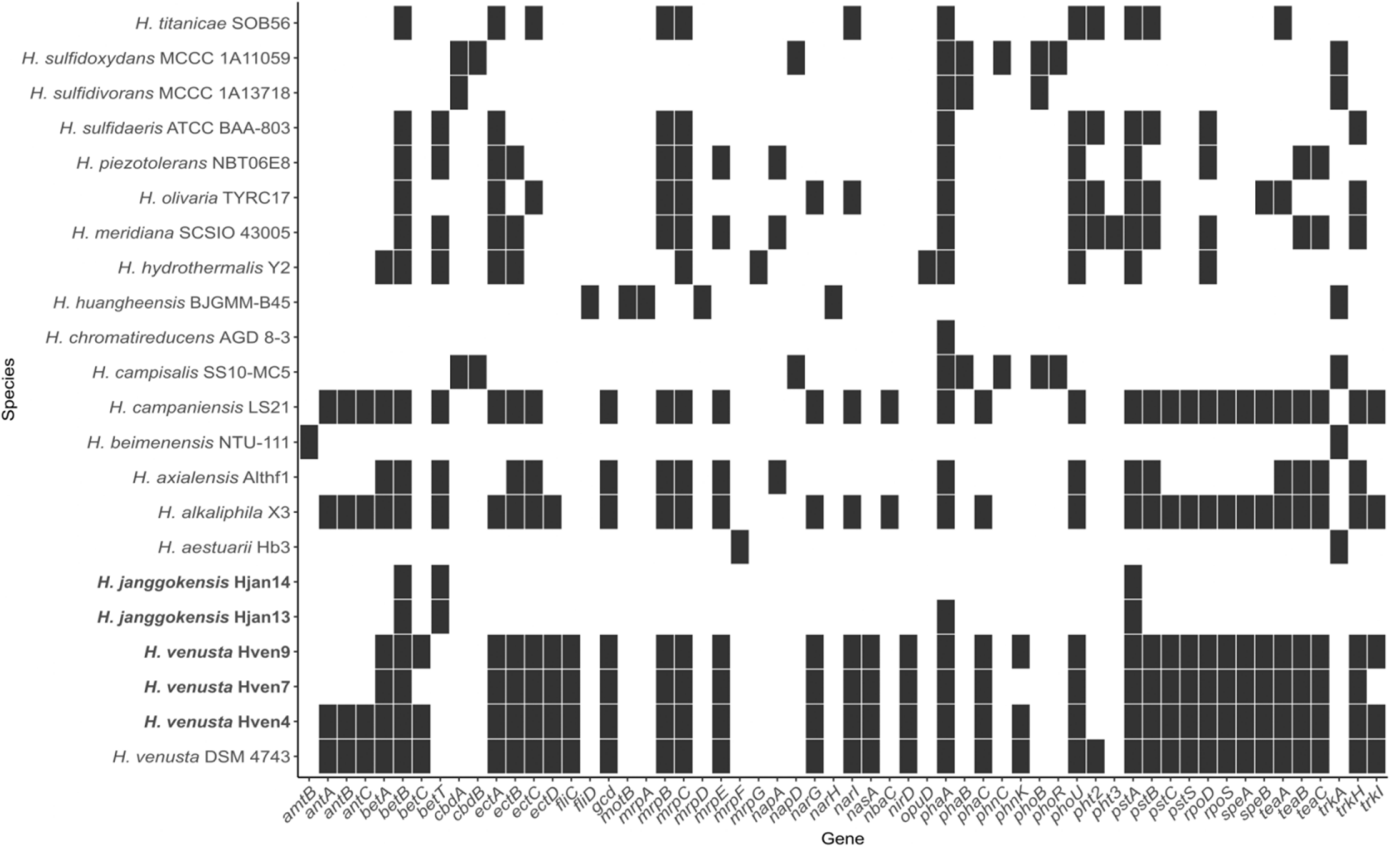
Heatmap of gene presence (black) and absence (white) of genes associated with the metabolism and homeostatic regulation of salt (*ect*ABC, *bet*ABC*,* and *trk*AHI), intake of nitrogen as nitrate and nitrite transporters (*nas*A, and *nar*GI), phosphorus uptake (*pst*ABCS*,* and *pho*BRU), transporters (*mrp*ABCDEF) and production of polyhydroxyalkanoates (*pha*ABC)

Most *Halomonas* species in this study presented a similar osmotic regulation strategy mediated by producing compatible solutes, with betaine and ectoine being the main osmoprotectants (Fig. 4). These molecules can be synthesized de novo or captured from the environment [66, 67]. Despite the clusters of genes involved in ectoine synthesis, *ect*ABC, and betaine, *bet*ABC, were present in most of the studied genomes. The ectoine transportation system *Tea*ABC was also detected in the *Halomonas* genomes. This transporter allows cells to accumulate suitable solutes when they are available in their medium [68]. *Halomonas* strains from Isabel Island showed a salt-out strategy related to the presence of the *ect*ABC and *bet*ABC gene cassettes*.* Although the biosynthesis of compatible solutes is energetically more expensive than other strategies [69, 70], it is not a problem in an environment that has a significant amount of nutrients available for intake, allowing the members of the community to deal with the annual changes in salinity mediated by the evaporation of water levels [6].

Strains in this study that did not present *ect*ABC nor *bet*ABC operons possessed the trk system that encodes Trk proteins responsible for K + ion intake. The Trk system requires ATP and drives potassium uptake through the transmembrane electrochemical proton gradient [71, 72], a mechanism of adaptation to higher saline levels [73]. Other mechanisms include the presence of the operon *mrp*ABCDEF, related to adaptation to osmotic pressures and pH homeostasis by changing salinity and pH via the efflux of monovalent cations such as K + , Na + , and protons) [74, 75].

### Nitrogen metabolism

**Fig. 5:**
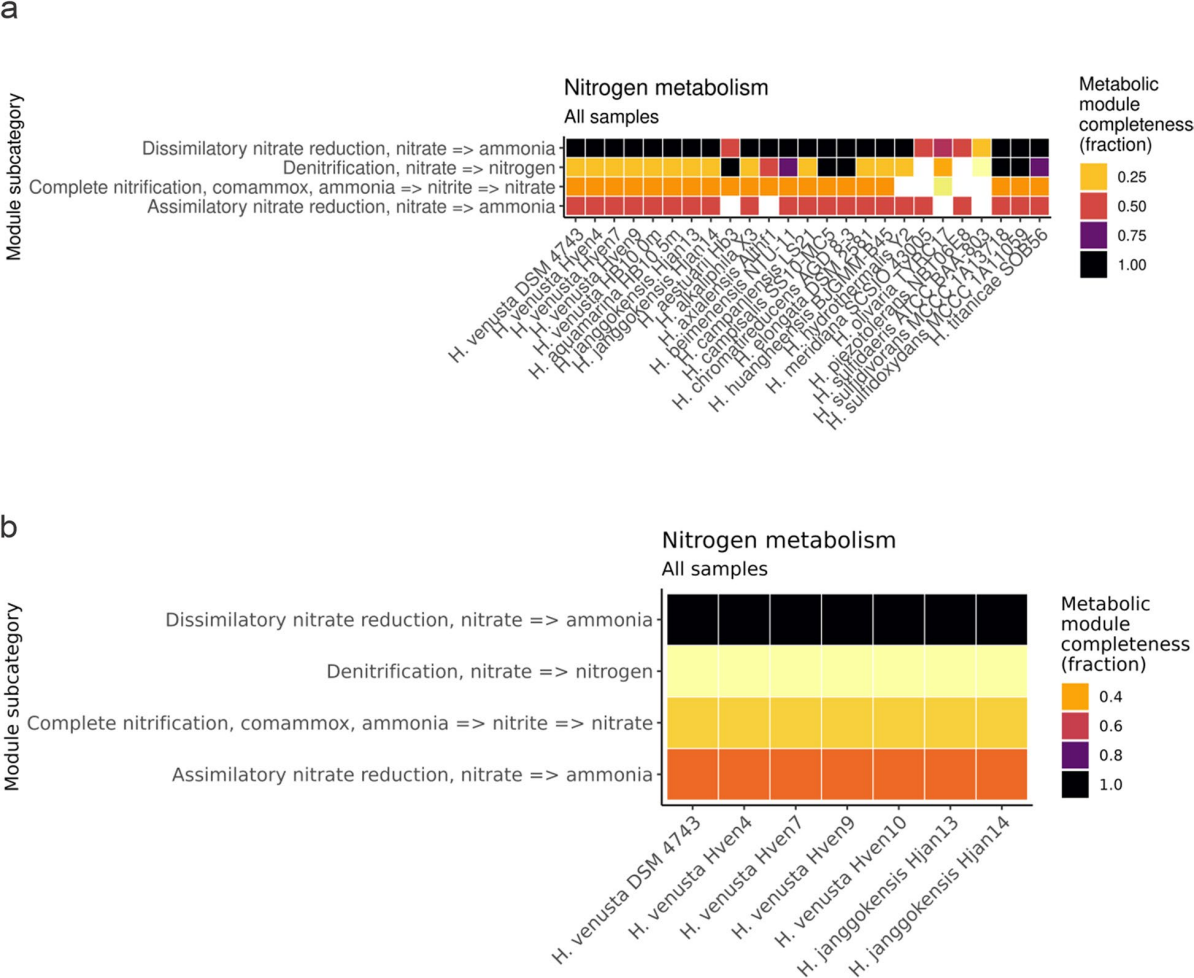
**A** Heatmap showing KEGG modules implied in the metabolism of nitrogen found in the complete genomes. Each row represents one KEGG Orthologous module. **B** Closeup to the nitrate metabolism among Halomonas isolated. Completeness higher than 0.70 is necessary to assume that the module has the number of genes to be considered entirely functional. Darker squares show more density of genes

*Halomonas* is known because of its variation in the inventory of denitrification genes [65]. Besides this, dissimilatory nitrate reduction is widely spread among the genus (Fig. 5a). A set of genes required for denitrification was found in the *H. venusta* strains, including membrane-associated nitrate reductase genes *nar*GH, nitrite reduction genes (either *nir*K or *nir*S) were not detected, but Isabel Island *Halomonas* have *nir*D presence, gene implied in that metabolism. The gene encoding periplasmic nitrate reductase *nap*A and its chaperone *nap*D were identified in some of the genomes. Assimilatory nitrate reduction is less present among the genus, but it could be detected. Nitrate reductase *nas*A was identified, but the genes encoding assimilatory nitrite reduction to ammonia (NIT-6 and *nir*A) were not identified in the genome. The inorganic nitrogen compounds acquisition and regulator gene *amt*B was present in only one of the genomes sets. The presence of this metabolic set can be related to the constant intake of seabird guano [1, 76]. Guano is rich in nitrogen in nitrate form [77]; the rapid action of the native bacteria explains the low levels of nitrate and high ammonia [6]. The presence of complete cassettes implied in dissimilatory nitrate reductions reinforces the importance of this compound for the metabolism of nitrogen in these domaining species (Fig. 5b).

### Phosphate metabolism

The intake of phosphorus by many bacteria is mediated by an ABC–transport complex, Pst (phosphate-specific transport), coupled to PhoU (a phosphate-specific transport system accessory protein) (Ma et al., 2016). It is an active transport system with a high affinity to Pi and has been well-characterized in Bacteria [78–80]. The Pst system is coded by the pstSCAB-phoU operon [81]. This system was detected in 15 of the genomes preserving the cassette in almost all of them, demonstrating the importance of facilitating and controlling phosphorus intake, as it is a limiting element, the genes implied in the intake of phosphorous are shown in Table 2.

**Table 2:** Genes implied in the phosphorous intake found in the strains Hven and Hjan

Gene	Function	Strain
phoU	Phosphate-specific transport system accessory protein PhoU	Hven4, Hven7, Hven9, Hven10
pstS_3	Phosphate-binding protein PstS precursor	Hven4, Hven7, Hven9, Hven10
pstS_2	Phosphate-binding protein PstS precursor	Hven4, Hven7, Hven9, Hven10
pstS_1	Phosphate-binding protein PstS precursor	Hven7, Hven9, Hven10
pstC_1	Phosphate transport system permease protein PstC 1	Hven7, Hven9, Hven10
pstC_3	Phosphate transport system permease protein PstC	Hven4, Hven7, Hven9, Hven10, Hjan13, Hjan14
pstA1	Phosphate transport system permease protein PstC	Hven4, Hven7, Hven9, Hven10, Hjan13, Hjan14
pstA	Phosphate transport system permease protein PstA	Hven4, Hven7, Hven9, Hven10, Hjan13, Hjan14
pstB3	Phosphate import ATP-binding protein PstB 3	Hven4, Hven7, Hven9, Hven10, Hjan13, Hjan14
pstB	Phosphate import ATP-binding protein PstB	Hven4, Hven7, Hven9, Hven10

As with nitrogen cycling, the seabird feces in Isabel Island form a eutrophic system with a large amount of available phosphorus in different forms. *H. venusta* strains have all the operon; meanwhile, *H. janggokensis* only some of the genes implied in the intake.

The utilization of phosphorus by specific members of the genus has been previously documented. Employing this capability for bioremediation to address highly eutrophicated waters, with a distinct emphasis on the removal of nitrogen and phosphorus, has yielded promising outcomes [82–84]. For this removal process, halophilic conditions and an alkaline pH (above 8.3) were necessary, which is consistent with Laguna Fragatas due to its alkaline pH [85].

### Differences between *H. venusta* and *H. janggokensis*

**Fig. 6:**
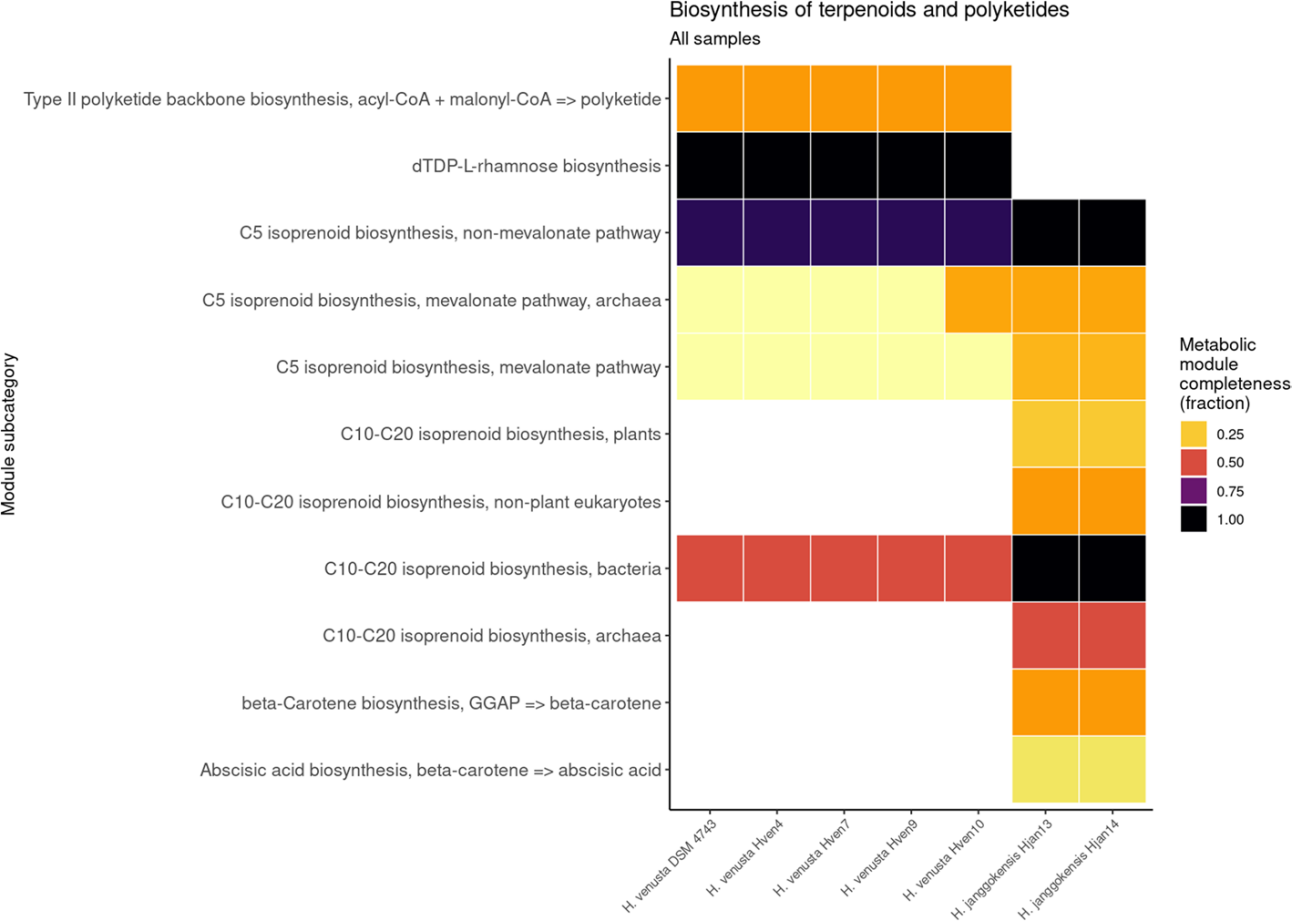
Heatmap showing KEGG modules implied in the metabolism of polyketides and isoprenoids found in the complete genomes of the Laguna Fragatas isolates

Other notable differences between *H. venusta and H. janggokensis* are associated with dTDP-L-rhamnose biosynthesis (Fig. 6), previously identified in a strain of *H. beimeninsis* [72]. Although this compound has been linked to the motility of pathogens, neither of the two species (*H. venusta or H. beimeninsis*) has been reported as pathogenic. However, it is worth noting that dTDP-L-rhamnose can serve as a substrate for the synthesis of rhamnolipids, which have biosurfactant capabilities [86]. *H. janggokensis* strains show complete cassettes related to C10-C20 isoprenoids, these compounds have been synthetized using microorganisms because of higher efficiency and more environmental friendliness than traditional plant extraction and chemical synthesis methods, improving the implemented for isoprenoid production in industry in the past few decades [87].

**Fig. 7:**
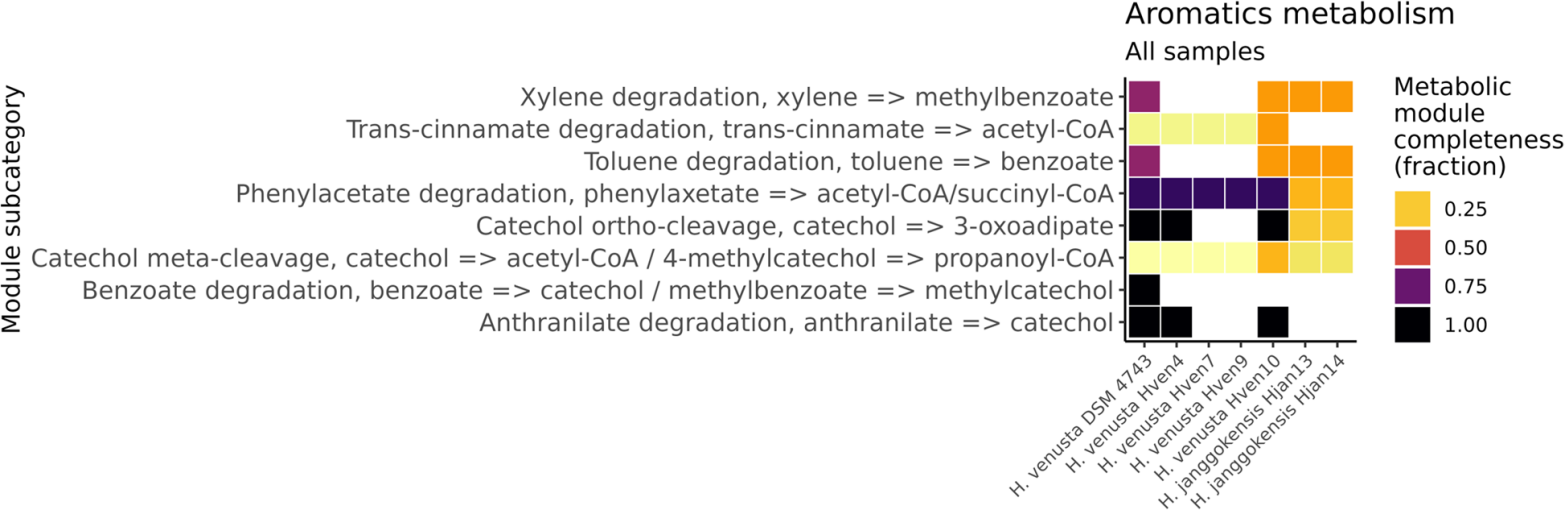
Heatmap showing KEGG modules implied in the metabolism of aromatic compounds found in the complete genomes of the Laguna Fragatas isolates

Hven4 and Hven10 show some interesting biosynthetic capabilities related to aromatics compounds (Fig. 7) like the genes related to the biotransformation of anthranilate into catechol. These have been reported in *E. coli* expressing *P. aeruginosa* genes [88]*.*

## Conclusions

In many saline environments members of the genus *Halomonas* are considered minor components of the community [89, 90] the study of Laguna Fragatas, an environment dominated by this family [26] gives a novel approach towards a non-typical hypersaline environment and its community dynamics; highlighting its capacity as a genetic, and biotechnological reservoir. This report compares the relationships between high-quality genomes of *Halomonas* and genomes of strains isolated from Laguna Fragatas using genomic, phylogenomic, pangenomic, and metabolic approaches, revealing spread similarities in metabolic adaptations and differences related to aromatic compounds metabolisms, isoprenoid biochemistry and phosphorous intake that make the genus highly adaptable to halophilic environments.

This analysis allows us to conclude that several genes are associated with metabolism and homeostatic regulation shared by all the species of *Halomonas* studied. This fact, particularly in Laguna Fragatas isolates, indicates that the adaptation to high concentrations of salt, nitrogen, and phosphorus uptake are highly optimized, as could be expected from an extremophile environment characterized by no disturbances or abrupt variations in the values of ecosystem parameters. Therefore, it is a result consistent with the presence of stable microbial communities, as has already been reported in other works for archaea, viruses, and bacteria [91, 92]**.**

In addition, this study is the first to analyze complete genomes of isolated bacteria from Isabel Island maar. It provides exploratory results about its metabolic potential, encouraging further preservation of the area and future research related to the production of metabolites of biotechnological interest, such as PHAs production. In this sense, the results of this study are consistent with other works where the relevance of the diversity of enzymes and metabolic pathways with biotechnological interest is found in this type of extreme environment. Finally, this work also reports the first two draft genomes of *H. janggokensis* strains.

### Supplementary Information

**Additional file 1: Supplementary Fig. 1.** Graphical representation of the genomes of Halomonas indicating from innermost ring: distribution of the GC content (black), GC skew (purple/green), and represented with different colors, the homology with the species within the genus. Currently, Halomonas elongata is recognized as the type species among the genus. Because of this, the comparison was made against its genome.

## Data Availability

Genome sequences of Halomonas venusta, strains Hven4, Hven7, Hven9, Hven10 have been deposited in GenBank under accession numbers CP090952 (https://www.ncbi.nlm.nih.gov/nuccore/CP090952.1), CP090951 (https://www.ncbi.nlm.nih.gov/nuccore/CP090951.1), CP090950 (https://www.ncbi.nlm.nih.gov/nuccore/CP090950.1), JAUYZU000000000 (https://www.ncbi.nlm.nih.gov/nuccore/JAUYZU000000000.1/) respectively; while genome sequences of *Halomonas janggokensis*, strains Hjan13 and Hjan14 have accession numbers JAKNQU010000001 to JAKNQU010000010 (https://www.ncbi.nlm.nih.gov/nuccore/JAKNQU000000000.1/), and JAKNQT010000001 to JAKNQT010000005 (https://www.ncbi.nlm.nih.gov/nuccore/NZ_JAKNQT000000000.1), respectively. The raw genomic data were deposited under SRA accession numbers SRR17542732, SRR17542733, SRR17542734, SRR17543027, and SRR17543028.
