# Supplementary figures and images for: Genome analysis of haloalkaline isolates from the soda saline crater lake of Isabel Island; comparative genomics and potential metabolic analysis within the genus Halomonas
Source: BMC Genomics. 2023 Nov 20;24:696. doi: 10.1186/s12864-023-09800-9 (PMC10662389; doi:10.1186/s12864-023-09800-9)

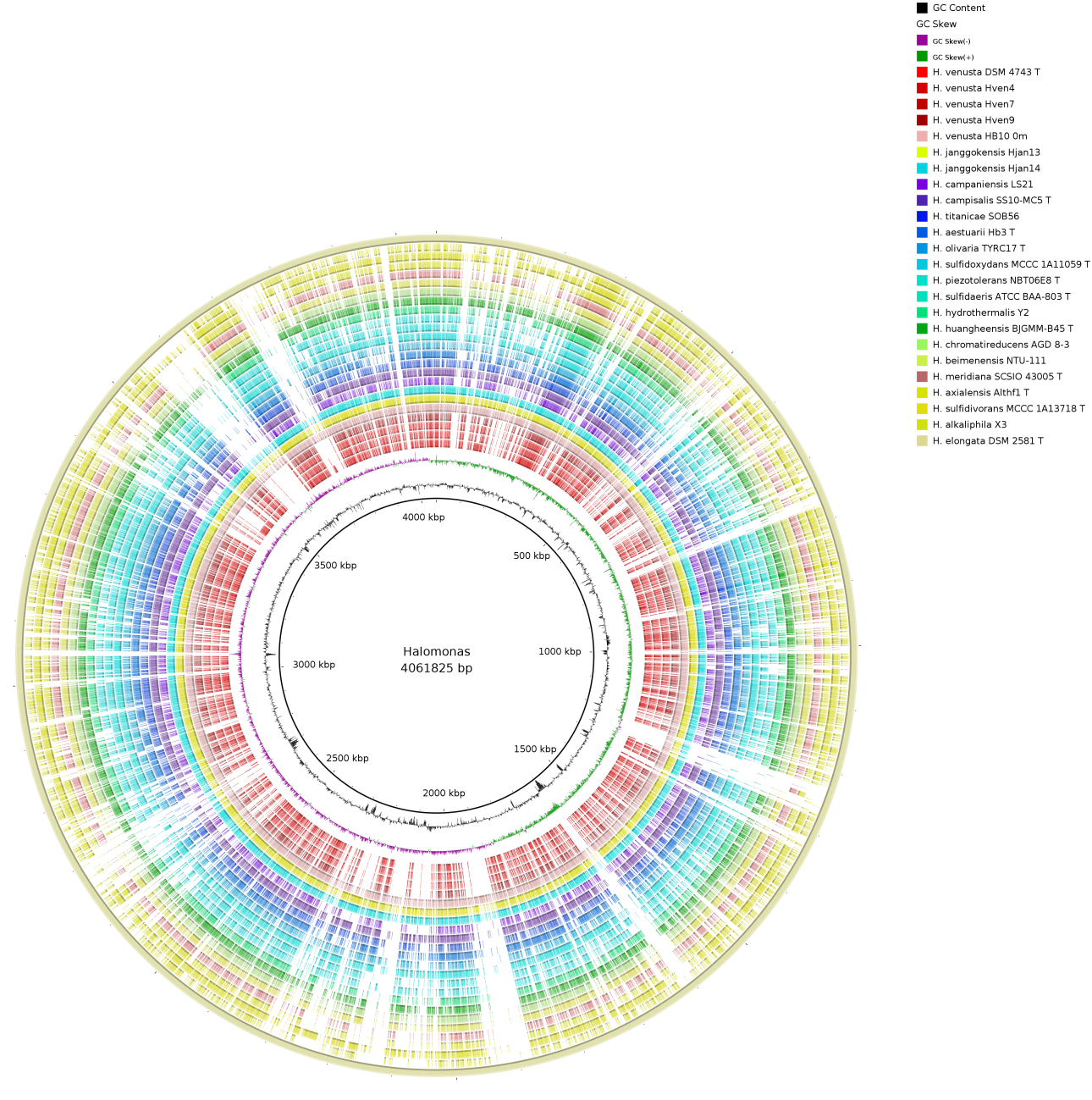

Supplement: Supplementary file 1 — Additional file 1: Supplementary Fig. 1. Graphical representation of the genomes of Halomonas indicating from innermost ring: distribution of the GC content (black), GC skew (purple/green), and represented with different colors, the homology with the species within the genus. Currently, Halomonas elongata is recognized as the type species among the genus. Because of this, the comparison was made against its genome. [file 12864_2023_9800_MOESM1_ESM.png]
